# Advancements in Cellulose-Based Superabsorbent Hydrogels: Sustainable Solutions across Industries

**DOI:** 10.3390/gels10030174

**Published:** 2024-02-29

**Authors:** Hossein Omidian, Arnavaz Akhzarmehr, Sumana Dey Chowdhury

**Affiliations:** Barry & Judy Silverman College of Pharmacy, Nova Southeastern University, Fort Lauderdale, FL 33328, USA; aa3853@mynsu.nova.edu (A.A.); sd2236@mynsu.nova.edu (S.D.C.)

**Keywords:** cellulose-based superabsorbents, sustainable agriculture, biodegradable hydrogels, environmental pollution control, eco-friendly construction materials

## Abstract

The development of superabsorbent hydrogels is experiencing a transformative era across industries. While traditional synthetic hydrogels have found broad utility, their non-biodegradable nature has raised environmental concerns, driving the search for eco-friendlier alternatives. Cellulose-based superabsorbents, derived from sustainable sources, are gaining prominence. Innovations include biodegradable polymer hydrogels, natural cellulose-chitosan variants, and cassava starch-based alternatives. These materials are reshaping agriculture by enhancing soil fertility and water retention, serving as potent hemostatic agents in medicine, contributing to pollution control, and providing eco-friendly construction materials. Cellulose-based hydrogels also offer promise in drug delivery and hygiene products. Advanced characterization techniques aid in optimizing their properties, while the shift towards circular economy practices further highlights sustainability. This manuscript provides a comprehensive overview of these advancements, highlighting their diverse applications and environmental benefits.

## 1. Introduction

The advent of superabsorbent hydrogels marks a transformative era in numerous industries, heralded by their unparalleled capability to absorb and retain substantial volumes of water and aqueous solutions. These hydrogels, traditionally synthetic in nature, have found widespread application in diverse fields, ranging from augmenting soil moisture in agriculture to enhancing comfort and efficiency in personal hygiene products like diapers [1,2,3,4,5,6,7,8,9,10,11].

Despite their utility, the environmental repercussions of synthetic superabsorbent hydrogels, predominantly due to their non-biodegradable nature, have raised considerable concerns. These materials contribute to persistent soil and water pollution, spurring a shift towards sustainable alternatives. The focus has turned to eco-friendlier options, such as hydrogels derived from natural polymers or biodegradable substances, which aim to match the absorbency of their synthetic counterparts while minimizing ecological impacts. Innovations in this area include the development of superabsorbent hydrogels using sustainable materials like sodium lignosulfonate and cellulose-based variants, which have shown promise in various industrial applications due to their impressive water absorption and retention capabilities [12,13].

The drive towards sustainability is further illustrated by the research into biodegradable materials like cellulose and carboxymethyl cellulose, which have demonstrated notable water absorption properties, signifying a leap towards environmentally benign solutions [14]. Additionally, cassava starch-based hydrogels offer a compelling alternative with their high-water absorbency and biodegradability, challenging the dominance of traditional synthetic polymers [15].

The burgeoning interest in sustainable materials like cellulose-based superabsorbent hydrogels is reshaping various sectors, including agriculture, medicine, environmental management, and construction. Their biodegradability, high water-absorption capacity, and versatility underscore their potential across a spectrum of applications. In agriculture, cellulose-based hydrogels have emerged as vital materials for enhancing soil fertility and water retention [14,16]. In the medical field, the biodegradable nature of these hydrogels has been leveraged for various applications, including as potent hemostatic agents to control massive bleeding [17]. Their role in environmental management, particularly in pollution control through the selective removal of oils and organic solvents, further highlights their significance [18]. Additionally, in construction and engineering, cellulose-based materials present eco-friendly alternatives to conventional products, as evidenced in the development of superhydrophobic aerogels for efficient oil absorption [19].

The broad applicability and eco-friendly nature of cellulose derivatives are central to the advancement of sustainable, high-performance materials. This is exemplified in the tailoring of smart and superabsorbent hydrogels, with cellulose-based materials playing a pivotal role [20,21]. The shift towards sustainable solutions is further emphasized by the exploration of the mechanistic roles of nanocellulose and cellulosic fibers in cellulose-based absorbents, underscoring their environmental benefits [22].

Innovation in material science is evident in the ongoing development and optimization of cellulose-based hydrogels [23]. As we look towards the future, the potential of cellulose-based superabsorbent hydrogels in various industries is immense, offering a pathway to more sustainable solutions.

This manuscript provides an overview of the latest advancements in the field of cellulose-based superabsorbent materials and slow-release fertilizers, highlighting their diverse applications across agriculture, the medical and hygiene sectors, environmental and pollution control, and construction and engineering. It underscores the development of innovative, eco-friendly, and sustainable solutions leveraging cellulose-based biopolymers and composites, aimed at improving resource efficiency, enhancing product performance, and addressing key environmental challenges. The focus is on the synthesis and optimization of these materials for various specific applications, from optimizing soil management in agriculture to developing advanced wound dressings in medicine, and from pollution mitigation to sustainable construction materials.

## 2. Cellulose-Based Superabsorbents for Soil Moisture and Nutrient Release

The following studies collectively focus on the development and application of cellulose-based superabsorbents and hydrogels in agriculture, particularly for enhancing soil moisture retention and nutrient release. These products, made from various cellulose derivatives and composites, are engineered to improve water absorbency, swelling properties, and thermal stability. Innovations include superabsorbent materials with additives like graphite oxide and bentonite, biodegradable hydrogels, and composites with enhanced mechanical strength. There is also a significant emphasis on coating fertilizers with these materials to extend nutrient release times and improve soil moisture retention. The research extends to creating environmentally friendly hydrogels and superabsorbent composites for slow-release fertilizers, demonstrating their potential in sustainable agricultural practices.

### 2.1. Cellulose-Based Superabsorbents and Hydrogels for Agriculture

Superabsorbent materials based on carboxymethylcellulose, enhanced with additives such as graphite oxide, reduced graphene oxide, activated carbon, and bentonite, were developed to improve gel properties. These superabsorbents underwent testing with electron beam radiation to study the impact of different additives, their quantities, and radiation types. They showed promising results in swelling tests in various solutions and demonstrated potential in agricultural applications, including with plants like spinach and crown daisy. Superabsorbents containing graphite oxide and reduced graphene oxide exhibited superior gel fraction and mechanical strength due to the strong binding between the polymer gel and carbon materials. These composites acted as slow-release liquid fertilizers, fostering improved growth in plants [24].

A composite superabsorbent polymer was created using alpha-cellulose, acrylic acid, acrylamide, and modified zeolite. Analyzed using techniques like FTIR spectroscopy, XRD, SEM, and TGA, this biodegradable composite exhibited enhanced water absorbency in both distilled water and a saline solution. Its improved water retention capacity and thermal stability suggest its usefulness in agricultural and horticultural applications [25].

A biodegradable polymer hydrogel was synthesized from water hyacinth cellulose, incorporating acrylic acid partially neutralized with ammonia. The process involved radical polymerization using N,N-methylene-bis-acrylamide as a crosslinker and ammonium persulfate as an initiator. This hydrogel’s swelling properties and biodegradability were significantly better than a copolymer without cellulose, indicating a substantial increase in the swelling capacity and degradation rate for agriculture applications [26].

Superabsorbent composites were synthesized using hydroxypropyl methylcellulose with acrylic acid, polyaspartic acid, and palygorskite. Created through aqueous solution polymerization, these composites, characterized by FTIR, SEM, and TGA, showed an optimal equilibrium absorption of 1785 g/g in deionized water. They significantly enhanced soil water holding and retention capacities and effectively reduced urea leaching and water permeability in soils, highlighting their potential for sustainable agricultural applications [27].

A superabsorbent hydrogel for agriculture was developed using carboxymethylcellulose sodium salt and hydroxyethyl cellulose, crosslinked with citric acid. This process integrated cellulose nanocrystals synthesized through acid hydrolysis. The influence of the cellulose nanocrystals on the hydrogel’s properties, including a 600% swelling rate with 2% citric acid as a crosslinker, was assessed. The focus was on optimizing crosslinkers and the distribution of nanocrystals within the hydrogel matrix to enhance water resource utilization in agriculture [28].

A biodegradable superabsorbent hydrogel from cellulose derivatives was developed, targeting the optimization of water resources in agriculture. This hydrogel’s sorption capability was evaluated in relation to its ionic strength and pH. Its effectiveness in efficiently storing and releasing water to soil and plant roots was demonstrated in experimental greenhouses with tomato cultivation, showcasing its potential in agricultural applications [29].

### 2.2. Fertilizer Coating and Release

**Figure 1:**
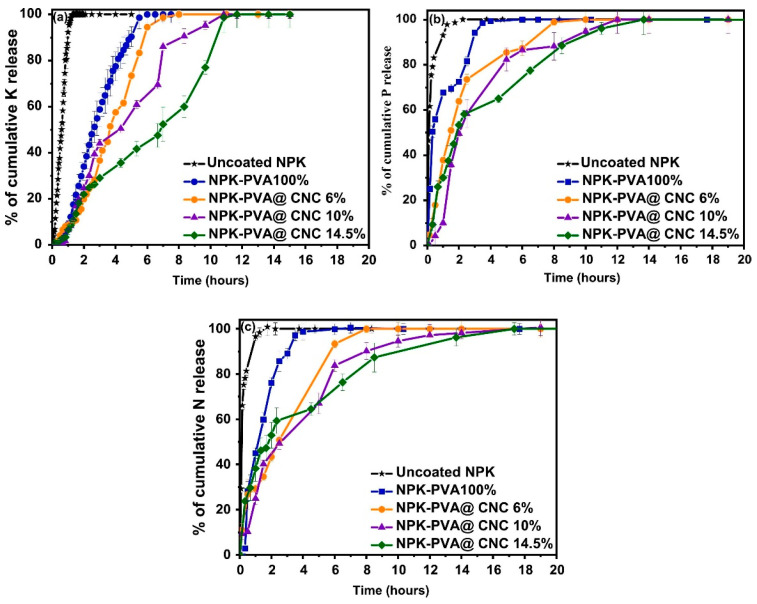
Release profiles of (**a**) potassium, (**b**) phosphorus and (**c**) nitrogen in a water medium from an uncoated and PVA@CNC-coated NPK fertilizer with different CNC loadings [30].

Polyvinyl alcohol (PVA) nanocomposites filled with cellulose nanocrystals (CNC), extracted from hemp stalks via sulfuric acid hydrolysis, were developed for coating NPK fertilizers. These nanocomposites, containing varying concentrations of CNC (6, 10, 14.5 wt%), were used to create a coating solution. The solution was uniformly applied to NPK fertilizer granules in the Wurster chamber of a fluidized bed dryer, under controlled conditions of spraying and drying. Studies on the coated fertilizers revealed that the coating significantly extended nutrient release and improved soil moisture retention, with these effects being more pronounced at higher CNC concentrations (Figure 1) [30].

A novel biodegradable cellulose hydrogel was formulated for coating a monoammonium phosphate (MAP) fertilizer. This hydrogel, comprising a blend of sodium carboxymethyl cellulose/hydroxyethyl cellulose and 5% spherical regenerated cellulose particles, was applied to MAP in a rotating pan. The resulting coated products, differing in thickness and crosslinking conditions, showed enhanced crushing resistance and better soil water retention. Release tests in both water and soil indicated that the coated MAP had a prolonged nutrient release compared to the uncoated version. It is suggested that the cellulosic materials in the primary coating could potentially double the nutrient release time of an MAP fertilizer and simultaneously improve soil moisture retention [31].

An innovative agrotextile material, designed for controlled fertilizer release and increased water absorption and retention, was also developed. This material involved coating nonwoven fabrics with a solution of carboxymethyl cellulose and potassium nitrate, using citric acid as a crosslinker for the cellulose. The coatings were applied to structures both with and without a prior polyvinyl alcohol coating. The characteristics of the coated materials, such as the gel fraction, water absorbency, and retention, were thoroughly examined. Additionally, the release of fertilizer from these structures was quantitatively assessed using a UV/Vis spectrophotometer [32].

### 2.3. Innovative Fertilizer Carriers and Controlled Release

A hydrogel based on cellulose was developed for the slow release of urea fertilizer. This involved modifying cellulose and integrating it with urea through crosslinking, followed by incorporating the fertilizer into the hydrogel. Its properties were characterized using FT-IR spectroscopy, elemental analysis, TGA, and SEM. The hydrogel exhibited effective swelling in various solutions, strong water holding and retention capacities, and showed potential for agricultural use in the controlled release of fertilizer [33].

Bacterial cellulose (BC) with a sphere-like structure was produced using Komagateibacter medellinensis derived from grape pomace, intended for use as natural fertilizer carriers. The production process was evaluated under both agitated and static culture conditions with different nitrogen sources, with agitation resulting in higher BC yields. The BC spheres showed a significant increase in their water-holding capacity and the ability to retain urea, up to 375% of their dry weight [34].

Research on controlled/slow-release fertilizers using cellulose biopolymers in composites was conducted, focusing on the surface chemical properties and hydrophilicity of cellulose. This research aimed at enhancing soil moisture retention and nutrient release in agricultural applications. It covered the modification of cellulose, its release mechanisms, and the potential impacts on sustainable agriculture, emphasizing the importance of eco-friendly, cost-effective technologies in promoting sustainability [35].

A superabsorbent composite for a slow-release urea fertilizer was synthesized through free-radical graft copolymerization. This process combined carboxymethyl cellulose, acrylamide, and montmorillonite, using N,N-methylenebisacrylamide as a crosslinker and potassium persulfate as an initiator. Analyzed using various spectroscopic and microscopic techniques, the composite demonstrated a high swelling capacity, which influenced the urea release rates, proving its effectiveness in controlled release systems [36].

A biodegradable superabsorbent composite made from cellulose and poly(lactic acid) was developed for agricultural applications. Designed to regulate water and fertilizer release, it was tested on Mediterranean crops in different soil types. The study focused on optimizing resource use through controlled solution release, analyzing the composite’s morphological, physical, chemical, and thermal properties [37].

Semi-interpenetrating polymer network (semi-IPN) superabsorbent resins with slow-release fertilizer capabilities were synthesized using corn straw cellulose polymer and linear polyvinyl alcohol (PVA). This involved extracting cellulose from corn straw using the nitric acid-aqueous solution method and incorporating ammonium polyphosphate (APP) for the nitrogen and phosphorus supply. The resin was characterized using SEM, FTIR, XPS, and TGA, showing excellent water absorbency, an enhanced soil water-holding capacity, and slow nutrient release [38].

Modified cellulose was utilized to create a superabsorbent with slow-release urea fertilizer properties. The modification involved converting cellulose to specific aldehyde and ene units, which were then used for eco-friendly superabsorbent synthesis. The resulting product’s properties, including its water absorbency, water retention, biodegradability, and slow-release capabilities, were extensively investigated, showing a significant impact on plant growth [39].

Novel controlled release fertilizer (CRF) beads were synthesized using sol–gel polymerization, with carboxymethylcellulose (CMC) as the matrix and kaolin as a binder. Iron (Fe^3+^) was used as a crosslinker to form spherical beads. The study optimized various parameters, such as the kaolin concentration and the crosslinking time and concentration. The urea-incorporated nanocomposite was confirmed through FT-IR, XRD, and SEM analyses, exhibiting optimal fertilizer release rates for different CMC concentrations [40].

Biodegradable hydrogel prototypes for slow-release fertilizers were formulated using a Schiff base reaction between dialdehyde carboxymethyl cellulose and gelatin. The release behavior of the iron cations from these hydrogels was studied, focusing on the correlation between the hydrogels’ correlation length and the degree of substitution. The structure and electrostatic interaction’s influence on cation release kinetics were analyzed, demonstrating the potential of the hydrogel with the lowest degree of substitution as a suitable matrix for slow-release fertilizers [41].

## 3. Biodegradable Hydrogels for Medical and Hygiene Applications

The focus of these entries is on developing biodegradable hydrogels for medical and hygiene applications, particularly in wound dressing, hemorrhage control, and personal care products. Utilizing cellulose-based materials, these hydrogels are engineered for a high absorption capacity, biocompatibility, and non-toxicity. Innovations include superabsorbent hydrogels for chronic wound dressings with enhanced water retention and a slow release, injectable hydrogel sponges for hemorrhage control with antibacterial properties, and modified bacterial cellulose for improved moisture balance in wounds. Additionally, the research encompasses the creation of eco-friendly, superabsorbent materials for personal care items like sanitary napkins, diapers, and drug delivery systems. These developments highlight the versatility and effectiveness of cellulose-based hydrogels in medical and hygiene sectors, offering sustainable and efficient solutions.

### 3.1. Wound Dressing and Hemorrhage Control

Cellulose-based superabsorbent hydrogels were created using cellulose from water hyacinth, treated with sodium hydroxide and urea. The addition of sodium tetraborate decahydrate (borax) as a crosslinking agent significantly improved the hydrogels’ absorption capacity, achieving up to 900% swelling. These hydrogels showed effectiveness against gram-positive bacteria and are being considered for use in wound dressings and as flame-retardant coatings [42].

A novel injectable hydrogel, QHM1, combining quaternized hydroxyethyl cellulose with mesocellular silica foam (MCF), has been developed for rapid hemorrhage control and wound healing. Through a one-pot radical graft copolymerization process, QHM1 exhibits a rapid expansion upon water contact, superior absorption, and promotes quick coagulation by concentrating blood components at the injury site. It also reduces plasma clotting time, provides a stable scaffold for platelet aggregation, and activates coagulation factors. Moreover, QHM1 demonstrates significant antibacterial properties and is compatible with living tissues, making it a promising candidate for clinical hemostatic applications due to its biocompatibility and biodegradability [43].

Bacterial cellulose (BC) underwent modification to improve its water retention after drying, aiming to create superabsorbent dressings for chronic wounds. This modification involved crosslinking BC with citric acid and various catalysts, including disodium phosphate, sodium bicarbonate, and ammonium bicarbonate, alone or in combination. The most effective combination, using disodium phosphate and sodium bicarbonate, resulted in a hydrogel with an increased water capacity and slower release compared to an unmodified BC. This enhanced BC is ideal for dressings in wounds with high amounts of exudate and is non-toxic, making it suitable for chronic wounds needing moisture balancing [44].

**Figure 2:**
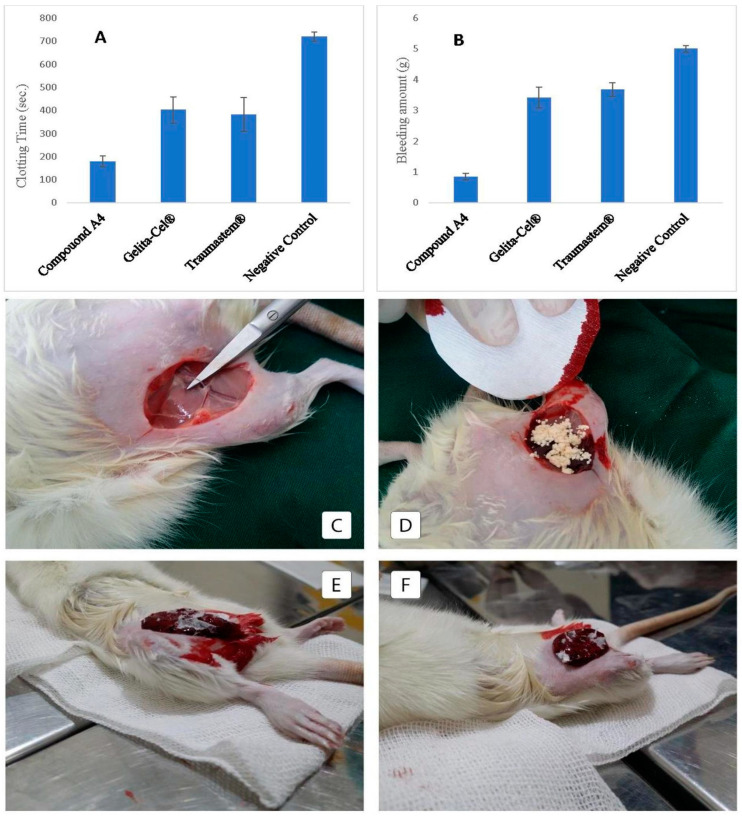
Evaluation of the performance of the most practical sample named as NDS (Compound A4) in severe bleeding due to the amputation of the rat femoral artery to record bleeding stop time and lost blood amount, with commercial Gelita-Cel^®^ and Traumastem^®^ powder as the positive control group and the untreated group as the negative control group. (**A**) Chart of clotting time, (**B**) Chart of bleeding amount (*n* = 5), and (**C**–**F**) images of bleeding position. (**D**) Related to commercial powder Gelita-Cel^®^, (**E**,**F**) related to Compound A4 (named as NDS) [17].

Novel developed superabsorbent (NDS) enriched with silica aerogel and calcium chloride, achieves superior hemostasis with a high absorption capacity (60 g/g) due to innovative crosslinking. Enhanced with quaternized hydroxyethyl cellulose and mesocellular foam silica (MCF), alongside silica nanoparticles (NPs), it rapidly activates the coagulation cascade, adheres to wounds, and forms a barrier for efficient blood absorption and clot formation. Proven safe and more effective than commercial alternatives in vivo, NDS and its complementary components offer a promising solution for fast bleeding control and wound healing (Figure 2) [17].

### 3.2. Biomedical Applications and Drug Delivery

An innovative, environmentally friendly method was used to develop a cellulose-based superabsorbent polymer (SAP). This involved transforming cellulose into carboxymethyl cellulose (CMC) using sodium monochloroacetate (MCA), followed by crosslinking with epichlorohydrin (ECH). The hydrogels produced showed a water retention value of 725 g/g in distilled water and a maximum of 118 g/g in a 0.9% NaCl solution, surpassing traditional cellulose-based SAPs. Analyzed using SEM, FTIR, and XRD techniques, these hydrogels displayed impressive re-swelling abilities, maintaining 90–95% of their original absorption capacity after four cycles of reuse. Their excellent swelling behavior in physiological saline positions them as strong contenders in hygiene and biomedical applications [45].

Cellulose-based hydrogels were developed for personal care products, featuring a three-dimensional network of cellulose chains. These superabsorbent materials, derived from plant or bacterial cellulose from Acetobacter xylinum, are designed to swell and absorb water and aqueous fluids while remaining insoluble. They are used as thickeners, stabilizers, and moisturizing agents in various products such as diapers, panty liners, tampons, paper towels, and tissue papers, offering different levels of absorbency [46].

A review focused on the grafting of hydroxyethylcellulose (HEC) with various polymers to create new biomaterials with improved properties for a range of applications. This process involves merging HEC, a cellulose derivative known for its biocompatibility and biodegradability, with polymers like polyacrylic acid and polyacrylamide. The resulting materials are suitable for drug delivery, hydrogels, superabsorbents, and personal hygiene products, highlighting their potential across various industrial sectors [47].

The development of superabsorbent sodium carboxymethyl cellulose (NaCMC) membranes for sanitary napkin was a key area of research. In this process, NaCMC was blended with starch and crosslinked using a unique combination of sodium trimetaphosphate (STMP) and aluminum sulphate (AlS). The production involved phase inversion (NaCMC-PI) and lyophilization, resulting in NaCMC/St-LY membranes with optimal crosslinking to prevent dissolution and disintegration. These biodegradable membranes, characterized by a microtextured surface and high water and blood absorption, retained about 50% of the water. They offer a sustainable alternative to non-biodegradable polyacrylate-based sanitary products [48].

## 4. Environmental and Pollution Control

The following studies focus on utilizing biodegradable cellulose-based hydrogels and superabsorbents for diverse environmental and pollution control applications. They encompass methods for detoxifying the production by-products, creating sustainable materials from agricultural waste, and synthesizing superabsorbents for biodegradability and water absorbency. Notably, the research includes developing hydrogels for heavy metal ion adsorption, dye pollutant removal, and oil spill cleanup. These hydrogels are characterized by their high absorption capacities, effective biodegradation, and potential for reducing environmental impact. Their applications range from cleaning up pollutants in water bodies to offering eco-friendly alternatives in various industries, demonstrating the versatility and environmental significance of cellulose-based materials in addressing pollution and promoting sustainability.

### 4.1. Environmental Sustainability and Detoxification Processes

An eco-friendly method was developed to detoxify washing waters from the production of biodegradable cellulose-based superabsorbent hydrogels. This process used divinylsulfone (DVS), a toxic crosslinker, in hydrogel preparation. The detoxification employed heterogeneous TiO_2_ photocatalysis, involving the irradiation of washing waters with suspended polycrystalline TiO_2_. This effectively removed the DVS, along with the unreacted polymers and oligomers. The efficiency of the photocatalytic process was assessed by measuring the total organic carbon (TOC) and monitoring the evolution of the sulfate anions [49].

Cellulose-based superabsorbent and flame-retardant aerogels were produced using blueberry tree pruning waste. These aerogels came in two forms: non-crosslinked (CA) and crosslinked with citric acid (CL-CA), both exhibiting a three-dimensional porous structure and excellent thermal stability. They had notable Brunauer-Emmet-Teller surface areas, 348 m^2^/g for the CA and 275 m^2^/g for the CL-CA. The production process was highlighted for its low cost and potential for industrial-scale production, emphasizing the value of these bio-wastes in creating future sustainable materials [50].

Superabsorbent hydrogels were synthesized from chitin and a cellulose/chitin mixture through esterification crosslinking using 1,2,3,4-butanetetracarboxylic dianhydride (BTCA). The chitin hydrogel, especially with a BTCA feed ratio of 5, displayed the highest water absorbency at 345 g/g-polymer. This method was also applied to cellulose/chitin mixtures, resulting in a 1:1 hybrid hydrogel with significant water absorbency (329 g/g-polymer). Both hydrogel types exhibited effective biodegradability when exposed to their respective enzymes [51].

### 4.2. Biomedical and Bioremediation Applications

A study focused on bacterial cellulose (BC), a versatile biomaterial, examining its production and diverse applications. BC is produced extracellularly by bacteria using renewable resources, resulting in a highly porous, biocompatible nanofibrillar structure. Its fine fibers and extensive porosity enable robust composite interactions and allow for a variety of chemical modifications. The review encompassed BC’s production and properties, highlighting its use in industries such as biomedical, food, paper, packaging, energy storage, and bioremediation. Additionally, the study explored BC’s potential in emerging technologies like battery separators and transparent displays, as well as its biomedical applications in wound healing, drug delivery, cancer treatment, cell culture, and artificial blood vessels. The role of additive manufacturing in creating complex scaffolds for biomedical purposes was also discussed [52].

A hydroxyethyl cellulose superabsorbent hydrogel, based on biomass lignin, was developed for dye pollutant removal. Constructed with long-chain hydroxyethyl celluloses as the backbone, short-chain polyvinyl alcohol as branched chains, and lignin molecules as crosslinkers, this hydrogel was created in an alkaline aqueous solution under gentle conditions. It exhibited a high swelling ratio (1220 g/g) and was effective in absorbing positively charged dyes like rhodamine 6G, crystal violet, and methylene blue. The hydrogels also demonstrated excellent water retention and biodegradability, making them suitable for use in commercial diapers, agriculture, and dye removal applications [53].

### 4.3. Heavy Metal and Pollutant Adsorption

Pectin/carboxymethyl cellulose-based hybrid hydrogels were synthesized for heavy metal ion adsorption, using epichlorohydrin (EPH) as a crosslinker. The study assessed the impact of crosslinker content on the hydrogels’ swelling behavior, observing the highest swelling at 8% (*v*/*v*) EPH at room temperature. Various analyses including FTIR, SEM, TGA, DSC, and AFM were conducted to evaluate the hydrogels’ morphology and thermal stability. Atomic absorption spectroscopy measured their adsorption capacity for different metal ion concentrations, and the Freundlich isotherm model was used to analyze the adsorption data. These hydrogels showed a high adsorption capacity for Cu, Pb, and Ni ions [54].

A lignocellulose-based superabsorbent polymer gel, crosslinked with magnesium aluminum silicate (MOT), was prepared for the removal of Zn (II) ions from water. Lignocellulose (LCE) underwent ultrasonic treatment and intercalation into clay to produce nano-LCE-MOT. Characterization methods included nitrogen adsorption/desorption, FTIR, SEM, and energy-dispersive X-ray spectroscopy. The adsorption kinetics followed a pseudo-second-order model, and the Langmuir isotherm model was applied. The gel demonstrated a maximum adsorption capacity of 513.48 mg/g, and optimal adsorption conditions and desorption methods were identified [55].

### 4.4. Oil Spill Cleanup

**Figure 3:**
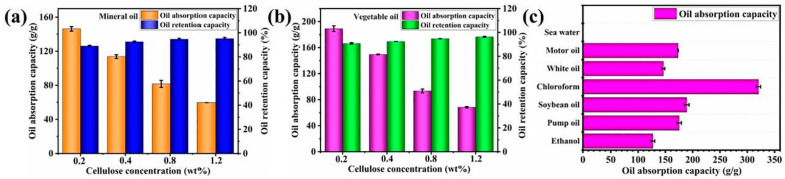
The oil absorption capacities and retention capacities of the KNFs for (**a**) mineral oil and (**b**) vegetable; (**c**) the absorption capacities of the 0.2 KNF for various oils and organic solvents [56].

A sustainable nanocellulose-based superabsorbent was developed from kapok fiber for oil spill cleanup. Kapok-derived cellulose nanofibril foams (KNFs) were produced with a hierarchically porous structure and an ultra-low density. These superhydrophobic KNFs excelled in oil absorption and oil–water separation (Figure 3). A novel oil release system was incorporated for easy reuse, with the Rigter–Peppas model confirming reusability through Fickian diffusion. The KNFs maintained their oil absorption and release effectiveness for at least 50 cycles of reuse [56].

## 5. Industrial and Construction Applications

The following studies collectively focus on the innovative use of cellulose-based materials in industrial and construction applications. They highlight advancements in enhancing cement and construction materials, like ordinary Portland cement and alkali-activated materials, with microcrystalline cellulose to modify the rheological and hardening characteristics. Research also delves into developing superabsorbent polymers (SAPs) for various industrial uses, using cellulose and starch derivatives, with an emphasis on improving water absorption and biodegradability. These developments underscore the versatility and potential of cellulose-based materials in improving industrial products and processes, aligning with sustainability and efficiency objectives.

### 5.1. Cement and Construction Materials

The influence of microcrystalline cellulose (MCC) on the properties of ordinary Portland cement (OPC) and alkali-activated material (AAM) was explored, focusing on aspects such as rheology, hydration kinetics, and early age strength under various curing conditions. The research involved incorporating different amounts of MCC, which led to enhanced viscosity recovery and thixotropic behavior in OPC matrices. In the AAM matrices, MCC significantly heightened the yield stress due to its gelation and swelling in NaOH. The presence of MCC in OPC-based matrices delayed the hydration process and reduced the heat release in the AAM after seven days. The early age compressive strength of the MCC-OPC matrices was observed to decrease with increasing MCC content. MCC was proposed as a potential additive for altering the rheological and hardening characteristics of OPC and AAM matrices [57].

A study was conducted to assess the impact of plant cellulose microfibers (CMFs), derived from kenaf strand fibers in two different sizes, on the hydration properties of cement composites. The CMFs were tested in proportions ranging from 0 to 2% of the cement’s weight. Key aspects evaluated included the setting time, heat of hydration, and compressive strength. Smaller CMFs were found to delay the setting and early hydration stages more significantly, while higher ratios of CMFs generally led to a reduced compressive strength. Notably, cement mixtures containing 0.3–0.6% CMFs demonstrated high strength and contributed to refining the pore structures of the composites [58].

Research was carried out on the development of a biodegradable nanocomposite, which involved grafting cellulose with butyl acrylate using emulsion polymerization without emulsifiers. This method used a transition metal complex initiating system comprising CuSO_4_, glycine, and ammonium persulfate, and incorporated kaolin to form the nanocomposite. The morphology of the resulting material was characterized using Fourier-transform infrared spectroscopy, X-ray diffraction, and field emission scanning electron microscopy. The nanocomposite exhibited improved thermal and mechanical properties. Its fire-retardant capabilities were evaluated using the limiting oxygen index and cone calorimetry tests. The biodegradation and water absorbency of these nanocomposites were also examined [59].

### 5.2. Superabsorbent Polymers for Industrial Uses

Superabsorbent polymers (SAPs) based on starch aldehydes and carboxymethyl cellulose were engineered, with starch aldehydes produced through periodate oxidation. This process formed acetal bridges in the SAPs, leading to enhanced water absorption capabilities. Citric acid, employed as a crosslinker, created ester bridges affecting water penetration. The swelling behavior of these SAPs was thoroughly analyzed using Fickian diffusion and Schott’s pseudo-second-order kinetics models. FE-SEM images were used to examine relationships between swelling and morphology, with the highest equilibrium swelling ratio recorded at 87.0 g/g. This result underscored the successful development of polysaccharide-based SAPs [60].

Sodium carboxymethyl cellulose/starch/citric acid SAPs were synthesized, utilizing citric acid and starch as eco-friendly crosslinking agents. These SAPs were optimized for maximum water absorbency, achieving 287.37 g/g in distilled water and 52.18 g/g in a saline solution. Swelling kinetics were assessed using Schott’s pseudo-second-order model. Additionally, the hydrogels underwent cytotoxicity testing and interaction analysis with the DNA Gyrase enzyme. The study highlighted that a small addition of citric acid to SAPs could significantly enhance their swelling rates [61].

Spherical, biodegradable hydrogel particles were created from sodium carboxymethyl cellulose (CMC) using ethylene glycol diglycidyl ether as a crosslinker. The synthesis process involved varying the initial concentration of CMC and the amount of ethylene glycol diglycidyl ether (EGDE) to control the particles’ shape, water absorbency, and enzyme degradability. The reaction mixture was introduced into fluid paraffin, with stirring-induced shear force forming spherical hydrogel particles. These particles were degradable by cellulase, making them suitable for various industrial and agricultural uses [62].

A superabsorbent derived from bacterial cellulose (BC) was synthesized via in situ fermentation on bentonite inorganic gel (BIG). The preparation of BIG was optimized by varying parameters such as the type and content of the sodium agent, the temperature, time, and the gelling agent type and content. The polymerization process took into account factors like the monomer/substrate ratio, initiator and crosslinker content, neutralization degree, and reaction conditions. The resulting optimal composite exhibited high water, salt absorption, and retention capacities. Characterization methods like XRF, NMR, FT-IR, SEM, and TGA were employed to assess water absorption and thermal stability [63].

A biodegradable superabsorbent polymer was developed from maleylated cotton stalk cellulose (MCSC) and acrylic acid (AA) using UV photopolymerization in an aqueous solution at room temperature. Irgacure 651 served as the photoinitiator. Characterization techniques such as FT-IR, H-1 NMR, SEM, and TGA were used to analyze the superabsorbent. The study evaluated how preparation conditions like the degree of substitution, MCSC amount, exposure time, photoinitiator amount, and monomer concentration affected the water absorbency and monomer conversion. The swelling kinetics, salt resistance, water retention, and biodegradability of the superabsorbent were also investigated [64].

A superabsorbent hydrogel was created using oil palm empty fruit bunch (EFB) cellulose and sodium carboxymethylcellulose (NaCMC) in a NaOH/urea system, with epichlorohydrin (ECH) as a crosslinker. This hydrogel’s swelling capacity exceeded 80,000% based on the NaCMC concentration and showcased rapid water absorption. ATR-FT-IR spectroscopy confirmed the formation of COC covalent bonds between dissolved EFB cellulose and NaCMC. The study also examined the swelling capacity and factors influencing the hydrogel’s physical and chemical properties [65].

Nanocomposite superabsorbent hydrogels (NCSHs) were prepared using carboxymethylcellulose (CMC) and various carbon materials, including graphite oxide (GO), reduced graphene oxide (rGO), and activated carbon. These were synthesized through electron beam radiation-assisted polymerization. Fourier-transform infrared spectroscopy and optical microscopy were used to characterize the structure and morphology, revealing well-dispersed carbon within the CMC matrix. The NCSHs demonstrated an improved mechanical strength and gel fraction compared to non-composite superabsorbent hydrogels. Swelling kinetics in different solutions indicated enhanced properties for the GO and rGO composites [66].

### 5.3. Specialized Applications

A cellulose-based superabsorbent polymer composite (SAPC), named Poly(acrylic acid-co-acrylamide-co-2-acrylamido-2-methyl-1-propanesulfonic acid)-grafted nanocellulose/poly(vinyl alcohol) composite (P(AA-co-AAm-co-AMPS)-g-NC/PVA), was developed specifically for the drug delivery of amoxicillin. The synthesis of this SAPC involved graft copolymerization. It was characterized using techniques such as FTIR, XRD, SEM, and DLS. Equilibrium swelling studies were conducted to evaluate its response to different stimuli, and the efficiency of drug encapsulation was determined at various amoxicillin concentrations. Drug release experiments were carried out in simulated gastric and intestinal fluids, following a non-Fickian mechanism, particularly at pH 7.4 [67].

Bio-based superabsorbent aerogels, made from cellulose nanofibers (CNFs), were developed with potential applications in baby diapers. These CNFs, TEMPO-oxidized to different degrees, were tested for their free swelling capacity (FSC). The results showed that the CNFs had a higher FSC compared to commercial fluff pulp and diaper absorbent materials, indicating these bio-based aerogels could be an effective alternative to traditional superabsorbent polymers in diapers. The study emphasized the biodegradability and sustainability of CNFs as key advantages [68].

A novel method to enhance cell deposition in 3D-bioprinted scaffolds was created by utilizing the superabsorbent properties of cellulose material-alginate hydrogels. Four different ink formulations were tested: 20:10 nanocrystal/alginate (NCA 20/10), 20:10 nanofiber/alginate (NFA 20/10), 20:02 nanocrystal/alginate (NCA 20/02), and 20:02 nanofiber/alginate (NFA 20/02). The scaffolds were designed with a dual-porous (DP) structure, connecting inherent pores (IPs) to the surface, which significantly improved cell absorption. This approach resulted in a more efficient cell deposition and a more uniform distribution of cells compared to traditional top-loading methods [69].

The study on the degradation of crosslinked carboxymethyl cellulose (CLD-2) focused on its interaction with various microbes and beta-glucosidase, particularly in relation to toxic shock syndrome. The findings revealed that none of the tested bacterial and yeast strains produced cellulases capable of hydrolyzing CLD-2, although all strains produced beta-glucosidase. This indicated that the role of vaginal microbes and beta-glucosidase in degrading CLD-2 in superabsorbent tampons, previously thought to be a contributing factor to toxic shock syndrome, should be re-evaluated [70].

## 6. Characterization and Analysis of Cellulose-Based Superabsorbent Hydrogels

Table 1 presents a comprehensive overview of how cellulose-based hydrogels are tailored for specific applications and how their properties are analyzed using various techniques. The correlation between the application of these hydrogels and their analysis is evident, with the chosen characterization methods directly related to the intended use of the polymer (see Table 2).

Table 2 provides an overview of the primary analysis and characterization techniques for cellulose-based hydrogels, aimed at optimizing their performance in agriculture, medical applications, environmental remediation, construction, and personal hygiene products.

Apart from the specific analysis and characterization procedures outlined in Table 2, there are general characterization techniques applied for further investigation into superabsorbent hydrogels (specifically those that are cellulose-based, as studied here). In spectroscopy, techniques such as Fourier-transform infrared spectroscopy (FTIR), X-ray diffraction (XRD), and UV–visible spectroscopy (UV–Vis) are emphasized. Microscopy applications involve the use of scanning electron microscopy (SEM), transmission electron microscopy (TEM), and atomic force microscopy (AFM). For thermal analysis, methods like thermal gravimetric analysis (TGA) and differential scanning calorimetry (DSC) are specified. Mechanical testing includes evaluations of the crushing strength, along with assessments of mechanical strength and flexibility. In the realm of chemical analysis, elemental analysis, nitrogen adsorption/desorption isotherms, and esterification crosslinking are mentioned. Lastly, biodegradability testing is addressed through enzyme degradability tests and assessments of biodegradability in soil, showcasing the diversity and applicability of these techniques across various scientific and engineering disciplines.

## 7. Development of Sustainable Superabsorbent Polymers

This analysis explores the development and effects of sustainable cellulose-based superabsorbent polymers (SAPs), focusing on their costs, environmental impacts, degradation behaviors, and a thorough cost–benefit review. It builds on the latest research to offer a well-founded perspective on the advantages and challenges these materials present.

Regarding production expenses, SAPs made from cellulose, especially those sourced from materials like flax yarn waste, go through intensive processing. This includes chemical changes and crosslinking to meet the required performance standards. Although these steps add to the overall expense, progress in technology and larger production scales are making these SAPs more cost-effective over time. Importantly, the initial higher costs are balanced by the long-term financial gains from using renewable resources and the possibility of composting, which presents a cost advantage over traditional petroleum-based SAPs [88].

Looking at the environmental side, SAPs made from renewable resources such as recycled waste fibers significantly lower the environmental toll. Their biodegradable nature is a stark contrast to the lasting environmental effects of synthetic SAPs, notably their role in microplastic pollution. The focus on using less harmful chemical processes in making cellulose-based SAPs underlines a strong commitment to reducing waste and emissions, furthering environmental protection efforts [89].

The biodegradability of cellulose-based SAPs is central to their environmental appeal. Their decomposition through enzymes and microbes supports sustainable environmental practices. Yet, finding the right balance between their biodegradability and the durability needed for certain uses presents a notable challenge. Ongoing research is aimed at finding the optimal balance to ensure these materials fulfill both functional and environmental standards effectively [45].

A detailed cost–benefit analysis of cellulose-based SAPs needs to consider their economic and environmental impacts. Despite the significant initial investment, the potential savings in waste management and environmental remediation costs are substantial. The environmental advantages, such as reducing the dependence on fossil fuels and cutting down carbon emissions, play a crucial role. However, it is vital to fully appreciate these benefits and challenges, including scaling up production and managing performance compromises, to have a well-rounded view [64].

In summary, cellulose-based superabsorbent polymers hold great promise for advancing sustainable material solutions. While there are obstacles related to cost and performance enhancements, their environmental benefits and support for sustainability goals highlight the necessity for ongoing research and development in this area.

## 8. Biodegradation of Cellulose-Based Superabsorbent Hydrogels

Materials such as cellulose, starch, and proteins are part of a group of natural materials that can biodegrade under suitable environmental conditions. This biodegradation is carried out by microorganisms like bacteria, fungi, and algae, which break down these materials into water, carbon dioxide, methane (in environments lacking oxygen), and biomass. The effectiveness of this process depends on the material’s chemical composition, environmental factors (temperature, humidity, and the presence of microorganisms), and any chemical alterations the material has undergone, such as crosslinking.

### 8.1. Crosslinking of Cellulose-Based Hydrogels

Crosslinking in cellulose-based hydrogels is an essential process for their production, converting cellulose into networks that absorb water. This process can be achieved through various techniques, each providing unique properties to the final hydrogel.

Physical crosslinking methods, including freeze–thaw cycles and ionic gelation, utilize non-covalent bonds [90,91,92]. The freeze–thaw technique involves cyclically freezing and thawing a cellulose solution, causing cellulose chains to aggregate. This method has been used to create cellulose–gelatin hydrogels with high strength and pH-responsive properties without using harmful crosslinkers [93]. Ionic gelation employs ionic crosslinkers, such as calcium ions, to form gels from cellulose solutions, resulting in soft hydrogels with excellent viscoelastic behavior [94,95,96,97,98].

Chemical crosslinking involves forming permanent bonds between cellulose molecules, leading to hydrogels with an improved mechanical strength and stability. This approach has been demonstrated to enhance the compressive properties, thermal stability, and hydrophobicity of cellulose nanofibril aerogels [99].

The sol–gel process dissolves cellulose in a suitable solvent to form a sol, which then transforms into a gel, facilitating the production of hydrogels with specific pore sizes and shapes. Various sol–gel methods for cellulose-based hydrogels have been reviewed, emphasizing the critical role of solvent systems and crosslinking techniques [100].

Supramolecular chemistry, which relies on non-covalent bonding, allows for the formation of hydrogels that can undergo reversible gelation and create hydrogels responsive to environmental stimuli. This field offers insights into the cellulose-based gels and microgels formed through these interactions, highlighting their potential in various applications due to their smart properties [101].

In conclusion, the crosslinking of cellulose-based hydrogels involves a variety of strategies that can be customized to achieve the desired characteristics in the final product, such as mechanical strength, biocompatibility, and a responsiveness to environmental changes. The selection of a crosslinking method, whether physical, chemical, or supramolecular, depends on the intended application and the required features of the hydrogel.

### 8.2. Crosslinking Cellulose-Based Hydrogels Using Biodegradable Crosslinkers

Cellulose-based materials are known for their ability to break down naturally, thanks to enzymes like cellulases. However, the process of making these materials stronger, more water-resistant, and more stable through crosslinking can slow down their breakdown. Crosslinking changes the structure of cellulose, making it harder for enzymes and microbes to decompose it. Despite this challenge, recent research has found ways to improve the properties of these materials while still keeping them environmentally friendly. For example, a study [102] on cellulose nanofiber-based bioplastics with double crosslinking showed a method that not only makes cellulose films stronger but also keeps them biodegradable. Another study [103] found that using natural crosslinkers in starch/cellulose nanocomposites can make them perform better without significantly reducing their ability to biodegrade, pointing towards more sustainable material improvements. Additionally, research [104] has introduced new crosslinking agents that are both biodegradable and compatible with living tissues, suggesting a balanced way to enhance material properties while protecting the environment. A further study [105] on regenerated cellulose with exceptional toughness indicates that advanced crosslinking techniques can create materials that are both mechanically superior and biodegradable, offering a path towards sustainable alternatives to plastics. Lastly, research [106] highlights the importance of choosing crosslinking substances and methods carefully to improve biopolymeric scaffolds without losing their biodegradability and non-toxicity, crucial for medical uses. Integrating crosslinking with cellulose-based materials appears to be an effective strategy to boost the physical qualities of biodegradable materials without greatly affecting their environmental benefits. By carefully selecting crosslinking agents and techniques, it is possible to develop cellulose-based materials that balance enhanced durability with biodegradability, meeting the needs for material strength and environmental protection.

## 9. Benefits of Using Sustainable Superabsorbent Polymers

Sustainable superabsorbent polymers (SAPs) are crucial in promoting sustainability, efficiency, and innovation across a variety of fields including agriculture, environmental management, medical advancements, and industrial innovations. Their extensive benefits play a key role in addressing significant challenges within these sectors.

In agriculture and soil management, cellulose-based SAPs significantly increase soil moisture content. This enhancement leads to a reduced need for irrigation and minimizes water wastage, supporting sustainable agricultural practices [24,25]. Moreover, SAPs improve plant nutrient delivery through controlled release technologies, which boosts crop growth and decreases fertilizer wastage [31,32]. The use of biodegradable hydrogels, made from renewable resources like water hyacinth and corn straw, reduces soil contamination and encourages the use of sustainable agricultural inputs [26]. Additionally, advancements in SAPs offer cost-effective solutions for managing moisture and nutrients in soil, making sustainable farming practices more accessible [27].

In the medical and hygiene sectors, SAPs have made significant contributions. For example, injectable and biodegradable hydrogels accelerate the healing process of wounds by promoting quick hemostasis and wound repair, representing a significant advancement in medical treatments [43,44]. The development of cellulose-based hydrogels for various medical applications indicates a commitment to sustainability without compromising on quality, making them suitable for sophisticated biomedical applications [42,45]. Furthermore, SAPs have enabled the creation of advanced biodegradable personal care products, improving comfort for users and significantly reducing waste, aligning with goals of environmental sustainability [48].

For environmental and pollution control, SAPs are vital in removing hazardous chemicals and pollutants like dyes and heavy metals from water, contributing to the cleanliness of water bodies and the overall health of the environment [49]. SAPs created from waste materials, such as flame-retardant aerogels, offer environmentally friendly solutions for environmental cleanup and emphasize the importance of repurposing waste [50,51]. Their use in managing oil spills, especially those based on nanocellulose, demonstrates their effectiveness in tackling major environmental issues [56].

In industrial and construction applications, integrating microcrystalline cellulose into construction materials enhances their durability and sustainability, leading to more environmentally friendly building practices [57,58]. Cellulose-based nanocomposites improve fire resistance and mechanical properties, essential for creating safe and durable construction materials [59]. SAPs also have wide-ranging uses in areas like drug delivery and 3D bioprinting, underscoring their vast potential. Research into their biodegradability and non-toxicity ensures these materials are used in a responsible and safe manner [64].

These contributions highlight the diverse advantages of sustainable superabsorbent polymers, emphasizing their importance in environmental sustainability, medical and hygiene innovation, agricultural efficiency, and industrial advancements. Their ongoing development and application provide a solid basis for future research and innovation in these crucial areas.

**Figure 4:**
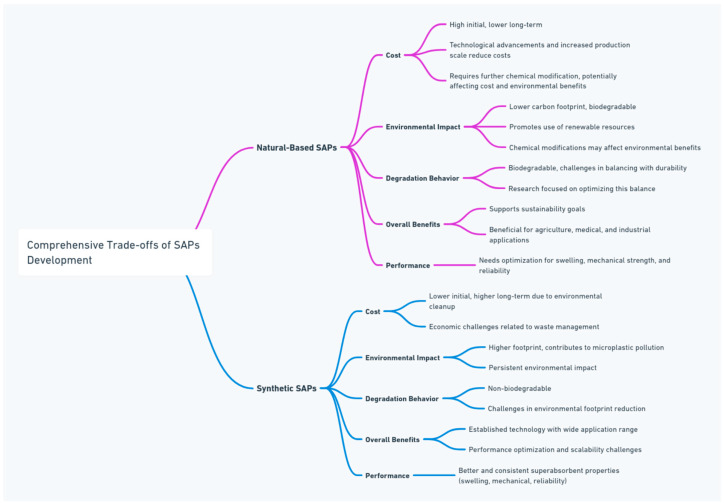
Trade-offs of developing SAPs.

Figure 4 illustrates the trade-offs in developing sustainable superabsorbent polymers compared with their synthetic counterparts.

## 10. Limitations in Using Sustainable Superabsorbent Polymers

The examination of sustainable superabsorbent polymers (SAPs) reveals a range of interconnected challenges and considerations, including material optimization, scalability, environmental impact, and the demands of specific applications. This comprehensive overview identifies key areas where further research and development are necessary.

Optimizing the components of SAPs is crucial for their performance. Achieving the right mix of cellulose derivatives, crosslinking agents, and additives is essential for improving their water absorbency, mechanical strength, and ability to respond to environmental changes. This is particularly important for their use in diverse fields such as agriculture and biomedicine [71,72,73,74]. Moving from laboratory-scale experiments to industrial-scale production introduces significant economic and technical challenges. These include making the production processes scalable, ensuring the commercial viability of new materials like water hyacinth cellulose, and maintaining the cost-effectiveness of producing cellulose-based SAPs and composites [26,27].

The potential environmental and health impacts of SAPs also require careful examination. It is necessary to study their biodegradability, the environmental consequences of their use, and the health risks posed by their components and their breakdown products. Assessing factors such as their long-term stability, how quickly they biodegrade, and the toxicity of certain additives, for example, borax, is crucial [25]. Moreover, advancing functionalization techniques to customize SAPs for specific applications is key. Tailoring properties like pore sizes, chemical structures, and responsiveness to environmental stimuli is vital for applications ranging from pollution control to personal care products [80].

It is also important to find the right balance between the mechanical strength and water absorption capacity of SAPs, particularly focusing on the equilibrium swelling ratio in carboxymethylcellulose-based superabsorbents. Optimizing formulations to enhance their effectiveness in agriculture and other areas requires adjusting the concentration of crosslinkers and the distribution of nanoparticles [24]. In addition, the practical application and field studies of SAPs, including their interaction with soil, effects on crop yield, and impact on soil health, warrant detailed investigation. Their performance in biological fluids and various environmental conditions highlights the need for adaptable and dependable solutions [29].

The efficiency of SAPs in removing pollutants varies depending on the type of contaminant, pointing to the necessity for optimization across a wide range of applications. The use of cellulose additives in cement composites and the environmental effects of materials like kaolin require specific approaches and additional research [54,55]. Finally, developing nanocellulose-based composites and other specialized SAPs often involves complex synthesis processes, high production costs, and safety concerns, particularly related to microbial interactions and the use of toxic crosslinkers. These factors may restrict their commercial viability and broader adoption [49].

Addressing these challenges demands a collaborative effort that combines expertise in material science, environmental studies, and application-specific research, aiming to advance the development and application of sustainable superabsorbent polymers.

## 11. Future Directions

The future development and application of sustainable superabsorbent polymers (SAPs) encompasses several key areas aimed at improving their environmental sustainability, performance, and economic feasibility.

The drive for innovation focuses on developing non-toxic, efficient crosslinking and synthesis methods to enhance SAPs’ performance while ensuring their biodegradability and environmental compatibility. Such advancements are critical for their broader adoption and application [25]. Additionally, there is a concerted effort to improve the mechanical properties, swelling capacity, and ionic sensitivity of SAPs through the integration of nanotechnology and novel material composites, aiming to expand their usability across various industries [81].

Understanding the environmental impact of SAPs throughout their lifecycle—from production to disposal—is essential for aligning with sustainability objectives. Comprehensive environmental impact assessments are required to evaluate their interactions with ecosystems and potential by-products [39]. Moreover, enhancing the scalability of production processes and conducting detailed economic analyses are pivotal for the commercial viability of sustainable SAPs. Innovations in manufacturing technologies and process optimization are necessary to lower costs and increase production efficiency [27].

Future strategies should also include the development of SAPs with customizable properties to meet specific environmental and agricultural needs, enhancing their practical application [37]. The integration of technological advancements and smart agriculture technologies promises to optimize SAPs’ water absorption capacity, nutrient release profiles, and mechanical strength [24]. Prioritizing the development of SAPs and cellulose-based hydrogels with improved biocompatibility and biodegradability is essential, especially for applications in medical and consumer products, to minimize potential toxicity and ensure safe degradation [42].

Transitioning promising hydrogels from laboratory research to clinical trials is crucial, necessitating demonstrations of safety and efficacy and navigating regulatory approvals [17]. There is also a need for SAPs capable of efficiently managing a range of biological fluids, with a focus on sustainable production processes and end-of-life disposal to minimize their environmental impact [45]. Enhancing SAPs’ versatility in pollutant removal through the development of materials with adjustable functional groups or composite materials for targeted contaminant removal is identified as a key research direction [54,55].

Comprehensive field trials and long-term studies are essential for evaluating the practical effectiveness, durability, and environmental impact of SAPs in real-world scenarios [53]. Encouraging interdisciplinary collaboration across fields such as chemistry, materials science, biomedical engineering, environmental science, and chemical engineering is vital for overcoming current challenges and maximizing the potential of SAPs in various applications [80]. Finally, engaging with regulatory bodies and policymakers to establish supportive frameworks is crucial for facilitating the market entry of sustainable SAPs and promoting the adoption of eco-friendly alternatives.

By addressing these focal points, the development of sustainable SAPs can make significant contributions to sectors including agriculture, healthcare, environmental remediation, and more, supporting global sustainability and environmental protection objectives.

## 12. Conclusions

The benefits of utilizing sustainable superabsorbent polymers (SAPs) are multifaceted and far-reaching, spanning the agriculture, healthcare, environmental management, and industrial sectors. These polymers play a pivotal role in enhancing soil moisture retention, promoting agricultural efficiency, and reducing water wastage. In healthcare, SAPs contribute to advanced wound healing and the creation of biodegradable personal care products, aligning with sustainability goals. Moreover, SAPs aid in environmental cleanup by removing pollutants from water bodies and mitigating oil spills. Despite their numerous advantages, challenges such as their material optimization, scalability, and environmental impact must be addressed through collaborative research efforts. Future directions include developing non-toxic synthesis methods, improving mechanical properties through nanotechnology integration, and conducting comprehensive environmental assessments.

## Figures and Tables

**Table 1:** Cellulose-based polymers, their specific applications, analyses, and characterization.

Cellulose Polymer Composition	Primary Application	Primary Analysis and Characterization	Ref.
Carboxymethylcellulose-based superabsorbents with additives like GO, rGO, activated carbon, and bentonite	Slow-release material for liquid fertilizers in agriculture	Gel strength, gel fraction, swelling tests, elution tests with urea, plant growth experiments	[24]
Alpha-cellulose and modified zeolite (MZE) based superabsorbent polymer composites	Water-keeping materials in agricultural and horticultural fields	FTIR spectroscopy, XRD, SEM, TGA, water absorbency and retention capacity tests	[25]
Water hyacinth cellulose-graft-poly(ammonium acrylate-co-acrylic acid) polymer hydrogel	Agriculture for biodegradability and effective utilization of water	FTIR, TEM, water absorption tests, degradation testing	[26]
Hydroxypropyl methylcellulose grafted with acrylic acid, polyaspartic acid and palygorskite	Improving water and fertilizer retention in soil	FTIR, SEM, thermal gravimetric analysis, equilibrium absorption tests	[27]
Carboxymethylcellulose sodium salt (CMCNa) and hydroxyethyl cellulose (HEC)-based hydrogels	Agriculture, for controlled water release	Fourier-transform infrared spectroscopy, SEM	[28]
Cellulose derivatives-based superabsorbent hydrogel	Water reservoir in agriculture and horticulture	Sorption capability assessment, experimental greenhouse cultivation, soil-water retention curve analysis	[29]
Cellulose nanocrystals (CNC) filled poly (vinyl alcohol) (PVA)	Waterborne coating materials for NPK fertilizer with slow release and water retention properties	Physico-chemical characteristics of CNC, morphology, coating rate, crushing strength, water-holding capacity and retention of soil	[30]
Biodegradable all-cellulose composite hydrogel from sodium carboxymethyl cellulose/hydroxyethyl cellulose blend with regenerated cellulose particles	Coating material for slow-release MAP fertilizer with water retention properties	Coating thickness, crosslinking conditions, crushing resistance, soil water retention capacity, release experiment of phosphorus and nitrogen	[31]
Carboxymethyl cellulose with potassium nitrate	Coating for nonwovens for efficient water and fertilizer management	Morphological and structural analyses, gel fraction, water absorbency and retention capacity, fertilizer release profile	[32]
Cellulose-based hydrogel modified and crosslinked with urea	Controlled release of fertilizer	FT-IR spectroscopy, elemental analysis, TGA, SEM, swelling behavior, water holding and retention behavior	[33]
Bacterial cellulose spheres biosynthesized from winery by-products	Natural carriers for fertilizers	Comparative study between agitated and static cultures, water-holding capacity (WHC) assays, fertilizer retention	[34]
Cellulose biopolymers as composite matrices	Controlled/slow-release fertilizers	Cellulose modification, slow/controlled-release mechanisms	[35]
Carboxymethyl cellulose-g-poly(acrylamide)/montmorillonite	Slow-release urea fertilizer	Fourier-transform infrared spectroscopy, X-ray diffraction, thermal gravimetric analysis, SEM, swelling capacity	[36]
Poly(lactic acid)/cellulose-based superabsorbent hydrogel composite	Water and fertilizer reservoir in agricultural applications	Morphological (SEM), physical (X-ray diffraction), chemical (EDX), and thermal properties (TGA, DSC)	[37]
Corn straw cellulose-based superabsorbent	Water-retaining and slow-release fertilizer	SEM, FTIR, XPS, TGA, water absorbency, slow-release performance	[38]
Modified cellulose as a grafting agent and flexible copolymer	Slow-release urea fertilizer	Different methods for characterizing modified cellulose, water absorbency, water retention capacity, reusability, biodegradability, slow-release property	[39]
Carboxymethylcellulose (CMC), Kaolin	Controlled release fertilizers (CRFs)	Sol–gel polymerization technique, FT-IR, XRD, SEM, UV–Vis Spectroscopy, Diacetylmonoxime method	[40]
Dialdehyde carboxymethyl cellulose and gelatin hydrogels	Biodegradable slow-release fertilizers	Schiff base reaction, study of release behavior of iron cations, Peleg’s Model	[41]
Cellulose-based hydrogels crosslinked with borax	Applications in wound dressing, agriculture, and flame-retardant coating	Swelling ratio analysis, antibacterial activity testing	[42]
Quaternized hydroxyethyl cellulose/mesocellular silica foam hydrogel	Hemostasis and wound healing	Radical graft copolymerization, water-triggered expansion, superabsorbent capacity, antibacterial activities, cytocompatibility	[43]
Crosslinked bacterial cellulose	Chronic wound dressings	BC crosslinking using citric acid with catalysts, water capacity and release testing, cytotoxicity tests	[44]
Cellulose-based polymer modified by silica aerogel and calcium chloride	Hemostatic agent	Chemical and physical crosslinking methods, blood absorption content, RBC attachment, blood clotting index, platelet adhesion, clotting time test, partial thromboplastin time, in vivo studies	[17]
Carboxymethyl cellulose (CMC) crosslinked with epichlorohydrin	Hydrogels for applications in hygiene and biomedical products	Water retention value measurement, compositional analysis, SEM, FTIR, XRD, re-swelling properties	[45]
Sodium carboxymethyl cellulose (NaCMC) and starch membranes crosslinked with sodium trimetaphosphate (STMP) and aluminum sulphate (AlS)	Absorbent core of sanitary napkins	Phase inversion and lyophilisation, water and blood sorption, mechanical strength and flexibility, biodegradability	[48]
Cellulose superabsorbent hydrogel	Preserve water in arid areas	Heterogeneous TiO_2_ photo-catalysed process, detoxification of washing waters, monitoring of total organic carbon and sulfate anions	[49]
Cellulose aerogel crosslinked with citric acid	Flame-retardant material	Brunauer–Emmet–Teller surface area measurement, thermal stability, flame-retardant performance	[50]
Chitin and cellulose/chitin-based superabsorbent hydrogels	Biodegradable products	Esterification crosslinking, water absorbency measurement, enzyme degradability by chitinase	[51]
Bacterial cellulose	Diverse applications including biomedical, food, paper, packaging, electrochemical energy storage	Production and properties review, potential for large scale applications, biomedical applications	[52]
Biomass lignin-based hydroxyethyl cellulose-PVA super-absorbent hydrogel	Dye pollutant removal	Swelling ratio, dye uptake capability, biodegradability, water retention	[53]
Pectin/Carboxymethyl Cellulose-based Hybrid Hydrogels	Heavy Metal Ion Adsorption	Solution polymerization, FTIR, SEM, TGA, DSC, AFM, atomic absorption spectroscopy (AAS), adsorption capacity analysis	[54]
Lignocellulose-Based Superabsorbent Polymer Gel Crosslinked with Magnesium Aluminum Silicate	Removal of Zn (II) from Aqueous Solution	Nitrogen adsorption/desorption isotherm, Fourier-transform infrared spectroscopy, SEM, EDX, adsorption kinetics and isotherm model analysis	[55]
Nanocellulose-Based Superabsorbent from Kapok Fiber	Oil Absorption and Recyclability for Spilled-Oil Cleanup	Porous structure analysis, oil absorption and release performance, Rigter–Peppas model, reusability assessment	[56]
Microcrystalline Cellulose in Portland Cement-Based and Alkali-Activated Slag-Fly Ash Blend	Construction Material	Rheology, hydration kinetics, early age strength analysis, micro-structural build-up	[57]
Plant Cellulose Microfibers in Cement Composites	Cement Composites Hydration	Hydration characteristics, setting time, heat of hydration, compressive strength, mercury intrusion porosimetry, scanning electron microscopy	[58]
Cellulose-g-poly(butyl acrylate)/kaolin Nanocomposite	Fire-retardant biomaterial	Emulsion polymerization, Fourier-transform infrared spectroscopy, X-ray diffraction, field emission scanning electron microscopy, thermal behavior, mechanical properties, fire-retardant properties	[59]
Superabsorbent Polymers based on Starch Aldehydes and Sodium Carboxymethyl Cellulose	Biodegradable superabsorbent polymers	FT-IR spectra, aldehyde quantitation, morphology in FE-SEM images, Fickian diffusion model, Schott’s pseudo-second-order kinetics model	[60]
Sodium Carboxymethyl Cellulose/Starch/Citric Acid Superabsorbent Polymer	Superabsorbent Polymer Production	FTIR analysis, TGA, water absorbency capacity, Schott’s pseudo-second-order model, cytotoxicity tests, in silico docking investigations	[61]
Spherical and Water-Absorbent Gels from Sodium Carboxymethyl Cellulose	Biodegradable Superabsorbent Polymer	Ethylene glycol diglycidyl ether as crosslinking agent, water absorbency, water-holding capacity, enzyme degradability	[62]
Bacterial Cellulose-Based Superabsorbent	Superabsorbent Production	Synthesis process optimization, water absorption, salts absorption, water retention capacity, characterization by XRF, NMR, FT-IR, SEM, and TGA	[63]
Maleylated Cotton Stalk Cellulose-g-poly(acrylic acid) Superabsorbent	Agriculture	UV photopolymerization, FT-IR, H-1 NMR, SEM, TGA, swelling kinetics, salt resistance, water retention, biodegradability	[64]
Oil Palm Empty Fruit Bunch Cellulose and Sodium Carboxymethylcellulose Hydrogel	Superabsorbent Hydrogel	ATR-FT-IR spectroscopy, crystallinity, thermal stability, gel fraction, swelling ability	[65]
Carboxymethylcellulose/Graphene Nanocomposite Superabsorbent Hydrogels	Superabsorbent Polymer and Additive Inorganic Nanomaterial	Electron beam radiation-assisted polymerization, Fourier-transform infrared spectroscopy, optical microscopy, mechanical strength, gel fraction, swelling kinetics	[66]
Poly(acrylic acid-co-acrylamide-co-2-acrylamido-2-methyl-1-propanesulfonic acid)-Grafted Nanocellulose/Poly(vinyl alcohol) Composite	Drug Delivery Vehicle for Amoxicillin	Graft copolymerization reaction, FTIR, XRD, SEM, DLS, equilibrium swelling studies, drug encapsulation efficiency, drug release kinetics	[67]
TEMPO-Oxidized Cellulose Nanofibers	Bio-Based Superabsorbent for Diaper Production	Free swelling capacity, comparison with commercial fluff pulp and diaper absorbent, suitability for baby diapers	[68]
Cellulose Material-Alginate Hydrogels	3D Bioprinting for Cell Deposition	Freeze-dried scaffolds, cell deposition, 3D bioprinting, cell viability	[69]
Crosslinked Carboxymethyl Cellulose	Toxic Shock Syndrome Research Related to Superabsorbent Tampons	Beta-glucosidase activity, microbial degradation, crosslinked cellulose analysis	[70]
Cellulose-Carboxymethyl Cellulose Beads	Hydrogel Beads for Various Applications	NMR relaxometry, bead-water interactions, swelling capacity, surface energy	[71]
Cellulose-Based Hydrogels Crosslinked with Citric Acid	Environmentally Friendly Hydrogels	DSC, FTIR, swelling measurements, reaction mechanism investigation	[72]
Hydroxyethylcellulose/Acrylic Acid Copolymer Gels	Superabsorbent Hydrogels	Radiation-initiated crosslinking, gel properties, electrolyte sensitivity	[73]
Carboxymethyl Cellulose Strengthened by TEMPO-mediated Oxidation Wheat Straw Cellulose Nanofiber	Hygienic and Horticultural Sectors	Citric acid crosslinking, swelling capacity, ionic sensitivity, biodegradability	[74]
Waste Hemicelluloses Lye for Superabsorbent Hydrogel Synthesis	High-Performance Superabsorbent Hydrogel	SEM, FTIR, TG, liquid absorbency, adsorption kinetics and isotherms	[75]
Cotton Cellulose and Succinic Anhydride Derived Superabsorbent Hydrogels	Biodegradable Superabsorbent Hydrogels	Esterification, absorbency in water and NaCl solution, biodegradability	[76]
Cellulose-Based Superabsorbent Hydrogels Crosslinked with Divinyl Sulfone	Degree of Crosslinking Evaluation of Hydrogels	C-13 solid state NMR, dynamic mechanical analysis, stress-deformation ratio, rubber elasticity theory	[77]
Cellulose-Based Microporous Superabsorbent Hydrogels	Personal Hygiene, Biomedical, and Industrial Applications	Chemical–physical structure analysis, equilibrium sorption properties, effect of crosslinking, pH, and ionic strength	[78]
Cationic Hydroxyethylcelluloses (PQ-4 and PQ-10) with Ethylenglycol Diglycidylether	pH-/Ion-Sensitive Drug Delivery Systems	Rheometric analysis, crosslinking kinetics, drug loading and release	[79]
Carboxymethylcellulose Hydrogels	High-Value Products via Circular Economy	Preparation and application of hydrogels, water absorption, mechanical strength, biodegradability	[80]
Regenerated Cellulose Products	Agricultural Applications	Dissolution–regeneration process, mechanical properties, potential in agriculture	[81]
Flax Shive Cellulose-Based Superabsorbent Polymer	Biodegradable Superabsorbent Polymer	Graft polymerization, SEM, FT-IR, TGA, biodegradability in soil	[82]
Nanocellulose Superabsorbent	Agricultural Growth of Spinach	Biodegradation, effect on soil properties and plant biomass, water management	[83]
Cationic Hydroxyethylcelluloses with Ethylenglycol Diglycidylether	Monitoring of Hydrogel Crosslinking Process	Ultrasonic wave propagation, dynamic mechanical analysis, acoustic behavior	[84]
Alginate-Carboxymethyl Cellulose Superabsorbents	Sustainable Superabsorbents for Various Applications	Quasi-cryogelation method, water absorption properties, morphology	[85]
Wheat Gluten Foams with Genipin and Cellulose Nanofibers	Superabsorbent and Biobased Protein Foams	Rapid and high water uptake, mechanical stability, capillary-driven absorption	[86]
Bacterial Cellulose Film from Cashew Apple Juice Processing Residue	Superabsorbent Biomaterial Production	FTIR, thermogravimetry, mechanical tests, water absorption capacity, SEM	[87]

**Table 2:** Primary analysis and characterization of cellulose-based hydrogels for their intended applications.

Field/Application	Specific Analysis and Characterization Techniques
Agriculture	Gel strength, gel fraction, swelling tests, elution tests with urea Water absorbency and retention capacity tests Plant growth experiments Equilibrium absorption tests Soil-water retention curve analysis
Medical Field (wound dressing, hemostasis)	Antibacterial activity testing Blood absorption content, RBC attachment, blood clotting index Cytocompatibility, cytotoxicity tests Radical graft copolymerization, chemical and physical crosslinking methods
Environmental Applications (dye pollutant removal, oil spill cleanup)	Dye uptake capability, biodegradability Oil absorption and release performance Adsorption capacity analysis for heavy metal ions
Construction Material	Rheology, hydration kinetics, early-age strength analysis Micro-structural build-up
Personal Hygiene Products	Phase inversion and lyophilisation Water and blood sorption tests

## Data Availability

Not applicable.
